# IL13Rα2 Is Involved in the Progress of Renal Cell Carcinoma through the JAK2/FOXO3 Pathway

**DOI:** 10.3390/jpm11040284

**Published:** 2021-04-08

**Authors:** Mi-Ae Kang, Jongsung Lee, Chang Min Lee, Ho Sung Park, Kyu Yun Jang, See-Hyoung Park

**Affiliations:** 1 Department of Biological Science, Gachon University, Seongnam 13120, Korea; makang53@hanmail.net; 2 Department of Integrative Biotechnology, Sungkyunkwan University, Suwon 16419, Korea; bioneer@skku.edu; 3 Department of Bio and Chemical Engineering, Hongik University, Sejong 30016, Korea; yycc456@naver.com; 4 Department of Pathology, Jeonbuk National University Medical School, Jeonju 54896, Korea; hspark@jbnu.ac.kr; 5 Research Institute of Clinical Medicine of Jeonbuk National University, Jeonju 54896, Korea; 6 Biomedical Research Institute of Jeonbuk National University Hospital, Jeonju 54896, Korea

**Keywords:** IL13Rα2, renal cell carcinoma, JAK2, FOXO3, telmisartan

## Abstract

Previously, we reported a close relationship between type II IL4Rα and IL13Rα1 complex and poor outcomes in renal cell carcinoma (RCC). In this study, we investigated the clinicopathologically significant oncogenic role of IL13Rα2, a kind of the independent receptor for IL13, in 229 RCC patients. The high expression of IL13Rα2 was closely related to relapse-free survival in specific cancers in univariate and multivariate analysis. Then, the oncogenic role of IL13Rα2 was evaluated by performing in vitro assays for cell proliferation, cell cycle arrest, and apoptosis in A498, ACHN, Caki1, and Caki2, four kinds of RCC cells after transfection of siRNA against IL13Rα2. Cell proliferation was suppressed, and apoptosis was induced in A498, ACHN, Caki1, and Caki2 cells by knockdown of IL13Rα2. Interestingly, the knockdown of IL13Rα2 decreased the phosphorylation of JAK2 and increased the expression of FOXO3. Furthermore, the knockdown of IL13Rα2 reduced the protein interaction among IL13Rα2, phosphorylated JAK2, and FOXO3. Since phosphorylation of JAK2 was regulated by IL13Rα2, we tried to screen a novel JAK2 inhibitor from the FDA-approved drug library and selected telmisartan, a clinically used medicine against hypertension, as one of the strongest candidates. Telmisartan treatment decreased the cell proliferation rate and increased apoptosis in A498, ACHN, Caki1, and Caki2 cells. Mechanistically, telmisartan treatment decreased the phosphorylation of JAK2 and increased the expression of FOXO3. Taken together, these results suggest that IL13Rα2 regulates the progression of RCC via the JAK2/FOXO3-signaling path pathway, which might be targeted as the novel therapeutic option for RCC patients.

## 1. Introduction

Every year, there are more than 300,000 new renal cell carcinoma cases (RCC) diagnosis globally [1]. Among them, about 30% of patients were diagnosed with metastatic RCC [2]. Moreover, the 5-year survival rate of patients with metastatic RCC is lower than 10% [3]. The prognosis of RCC patients is divided into several categories, such as favorable, intermediate, and poor-risk disease according to well-characterized clinical and laboratory risk factors [4]. Approximately 75% of patients with RCC have a poor-risk disease, and their prognosis is worse than that with a favorable-risk disease [5,6]. Over the past decade, there have been marked advances in the treatment of metastatic RCC. Sorafenib, sunitinib, bevacizumab, and axitinib are effective inhibitors of vascular endothelial growth factor (VEGF) and its receptor (VEGFR) [7]. Everolimus and temsirolimus inhibited the mechanistic target of rapamycin complex 1 (mTORC1) [8]. However, the mortality rate of metastatic RCC is still high because of resistance to conventional chemotherapy and the side effect of radiation therapy [9]. Therefore, we still need to consider the efficient treatment option for RCC.

IL13Rα2 is a membrane-bound protein encoded by the IL13Rα2 gene [10]. IL13Rα2 is closely associated with IL13Rα1, a subunit of type II IL4Rα and IL13Rα1 complex [10]. IL13 binds IL13Rα2 with high-affinity [10]. Recently, IL13Rα2 has been considered an important target for cancer treatment in various clinical studies [11]. A recent study indicated that IL13Rα2 is a potential marker and therapeutic target for human melanoma treatment [12]. It was reported that IL13Rα2 was overexpressed in metastatic colorectal cancer and inhibition of IL13 binding to IL13Rα2 showed the therapeutic activity in colorectal cancer by reducing metastatic spread [13]. Furthermore, it was demonstrated that targeting IL13Rα2 depletion suppressed breast tumor growth and IL13Rα2 activated IL13-mediated STAT6-signaling pathway, and knockdown of IL13Rα2 suppressed breast cancer metastasis into the lung [14]. Therefore, IL13Rα2 could be a potential biomarker to diagnose various cancers. However, there is not enough study for the clinical analysis, biological function, and molecular mechanisms of IL13Rα2 in RCC development.

Drug repositioning means the application of the drugs that have been clinically used to other diseases by elucidating the novel activities and target proteins [15]. Since the clinically used drugs were approved by the US Food and Drug Administration (FDA), drug repositioning has many advantages, such as no need to test toxicity and to evaluate pharmacokinetics. Furthermore, there are many previous reports and patents for studying the metabolism and interactions of the old drug, which could help researchers to examine the possible working mechanism of the old drug in the new application. Thus, drug repositioning could considerably save the cost and time for researchers to develop efficient drugs leading to improve the success rate [16]. For the proof-of-concept trial, we successfully selected telmisartan, a clinically used medicine against hypertension, as the strongest JAK2 inhibitor. Telmisartan is known as an agonist of angiotensin II receptor, but not reported on the possible involvement of-signaling pathway, including the regulation of JAK2 [17].

In this study, we investigated the clinical implication and oncogenic role of IL13Rα2 in RCC progression. Interestingly, IL13Rα2 seemed to increase the phosphorylation of JAK2 and decrease the expression of FOXO3. These results suggest that IL13Rα2 regulates RCC progression through JAK2/FOXO3-signaling pathway. Since JAK2 was regulated by IL13Rα2 and type II IL4Rα and IL13Rα1 complex, we tried to screen an FDA-approved drug library with a JAK2 kinase assay kit to identify the novel candidates that were possibly inhibiting JAK2 in RCC cells. Here, we show that telmisartan has the potential for antiproliferative activity in RCC cells, which could broaden the therapeutic options for RCC patients.

## 2. Materials and Methods

### 2.1. RCC Patients and Tissue Samples

RCC patients who operated between July 1998 and August 2011 at Jeonbuk National University Hospital were analyzed in this study. Medical records, histologic and tissue samples were available in 229 cases and included in this study. The clinicopathologic information for patients with RCC was obtained by analyzing medical records and original histologic slides. Tumor stage and histopathologic factors were re-evaluated according to the World Health Organization classification of the renal tumor [18] and the 8th edition of the staging system of the American Joint Committee on Cancer [19]. Histological subtypes of RCCs included in this study were 201 cases of clear cell RCC (CCRCC), 16 cases of chromophobe RCC, and twelve cases of papillary RCC. This study obtained institutional review board approval from Jeonbuk National University Hospital (IRB No., CUH 2019–11–039) and was performed according to the Declaration of Helsinki. The approval contained a waiver for written informed consent based on the retrospective and anonymous character of this study.

### 2.2. Immunohistochemical Staining and Scoring

Immunohistochemical staining in RCC tissue was performed using tissue microarray sections. One 3.0 mm core per case was arrayed in tissue microarray. The tissue microarray core was obtained from the area of the original paraffin-embedded tissue block, mainly composed of tumor cells with the highest histologic grade. The histologic sections were deparaffinized and boiled with the microwave oven for 20 min in pH 6.0 antigen retrieval solution (DAKO, Glostrup, Denmark) to induce antigen retrieval. Thereafter, the tissue sections are incubated with anti-IL13Rα2 primary antibody (1:100 dilution, Santa Cruz Biotechnology, Santa Cruz, CA, USA) and visualized using the enzyme-substrate 3-amino-9-ethylcarbazole. Immunohistochemical staining scoring was performed by two pathologists (HSP and KYJ) with consensus by observing in a multi-viewing microscope. The scoring was performed without clinicopathologic information. The score obtained by adding staining intensity point (point 0; no staining, point 1; weak, point 2; intermediate, point 3; strong) and staining area point (point 0; no staining, point 1; 1%, point 2; 2–10%, point 3:11–33%, point 4; 34–66%, point 5; 67–100%) [20,21,22]. Therefore, the immunohistochemical staining score ranged from zero to eight.

### 2.3. Chemical Reagents, Antibodies, and Plasmid DNAs

The FDA-approved drug library (SCREEN-WELL FDA-approved drug library V2, 821 drugs) was purchased from Enzo Life Sciences (Farmingdale, NY, USA). Mouse anti-β-actin antibody, mouse anti-Myc antibody, mouse anti-HA antibody, protease inhibitors, phosphatase inhibitors, AZD1480, telmisartan, the following chemicals, and solvents (non-fat dry milk powder, dimethyl sulfoxide (DMSO), ethylenediaminetetraacetic acid (EDTA), glycerol, glycine, sodium chloride, Trizma base, Triton X-100, sodium dodecyl sulfate (SDS), crystal violet, 4% paraformaldehyde solution, 4′,6-diamidino-2-phenylindole (DAPI), propidium iodide (PI), and Tween-20) were from Sigma (St. Louis, MO, USA). Control siRNA, siRNA against IL13Rα2, protein A or G-agarose beads, rabbit anti-IL13Rα2, and rabbit anti-FOXO3 antibodies were purchased from Santa Cruz Biotechnology (Santa Cruz, CA, USA). Rabbit anti-JAK2, rabbit anti-phospho-JAK2 (pJAK2), rabbit anti-cleaved PARP1, rabbit anti-cleaved caspase3, and rabbit anti-p27 antibodies were purchased from Cell Signaling Technology (Danvers, MA, USA). Goat anti-rabbit and goat anti-mouse horseradish peroxidase (HRP)-conjugated IgG (heavy/light or light chain-specific) were from Jackson ImmunoResearch (West Grove, PA, USA). Enhanced chemiluminescence (ECL) reagent was from GE Healthcare (Little Chalfont, United Kingdom). pCMV3-C-HA and pCMV3-JAK2-C-HA plasmid DNA were from Sino Biological (Wayne, PA, USA). pCMV6-C-Myc-Flag and pCMV6-IL13Rα2-C-Myc-Flag plasmid DNA were from OriGene (Rockville, MD, USA).

### 2.4. Cell Culture

A498, ACHN, Caki1, Caki2, and 293T cells were purchased from ATCC (Manassas, VA) and were grown in Dulbecco’s modified Eagle’s media (DMEM, Invitrogen, Carlsbad, CA, USA) media containing 10% fetal bovine serum (FBS, Invitrogen) and 1% streptomycin/penicillin. The cells were cultured in a humidified incubator (5% CO2, 37 °C). We performed all experiments with early passages cells (passages 4–10).

### 2.5. Transfection of siRNA and Plasmid DNA

Cells were plated (5.0 × 10^5^ cells/well) in 60 mm cell culture dishes and incubated for 18 h in an incubator. After 18 h of incubation, cells were transfected with siRNAs (siRNA against IL13Rα2: sc-63339, control siRNA: sc-37007 from Santa Cruz, 1 µL) or plasmid DNAs (pCMV3-C-HA empty/HA-JAK2 plasmid DNA, 1 µg). siRNAs or plasmid DNAs were mixed with 3 µL of lipofectamine 2000 (Invitrogen), respectively, in 600 µL of serum-free media for 20 min. After PBS washing twice, the cells were incubated with the media containing siRNAs or DNAs for 6 h in a humidified incubator. After 6 h, cell culture media was removed, and fresh media containing 10% FBS was added. After then, the cells were incubated for 18 h.

### 2.6. WST-1 Assay

Cells were plated (1 × 10^3^ cells/well) in 96-well plates and incubated for 18 h in a humidified incubator. After incubation, cells were transfected with control/IL13Rα2 siRNA or treated with DMSO (0.1%) control/the indicated treatment for 24, 48, or 72 h. After incubation, 20 µL of EZ-Cytox (DoGenBio, Republic of Korea) was added to the medium. After 4 h, absorbance was measured at 460 nm wavelength by a microplate reader (Bio-Rad Laboratories, Hercules, CA, USA).

### 2.7. Cell Counting Assay

Cells were plated (2 × 10^4^ cells/well) in 60 mm culture dishes and incubated for 18 h in an incubator. After incubation, cells were transfected with control/IL13Rα2 siRNA or treated with DMSO (0.1%) control/the indicated treatment for 14 days. The number of cells was counted by a hemocytometer.

### 2.8. Colony Formation Assay

Cells were plated (5 × 10^2^ cells/well) in 60 mm culture dishes and incubated for 18 h in a humidified incubator. After incubation, cells were transfected with control/IL13Rα2 siRNA or treated with DMSO (0.1%) control/the indicated treatment for 2 weeks. Cells were transfected with IL13Rα2 or control siRNA every other day, changing cell culture media. Similarly, Cells were treated with telmisartan or the same volume of DMSO vehicle every other day, changing cell culture media. The cells were fixed with 4% formaldehyde (Sigma) and stained using 1% crystal violet (Sigma). The number of colonies was counted.

### 2.9. Cell Cycle Analysis

Cells were plated (5 × 10^5^ cells/well) in 60 mm cell culture dishes and incubated for 18 h in a humidified incubator. After incubation, cells were transfected with control/IL13Rα2 siRNA or treated with DMSO (0.1%) control/the indicated treatment for 48 h. Then, the cells were trypsinized and fixed in 70% ice-cold absolute ethanol overnight at −20 °C. After then, centrifugation was carried out (1000 rpm, 5 min), and the cells were suspended with propidium iodide (PI) solution for 30 min at 37 °C. After staining, cell cycle distribution was analyzed by a FACSCalibur (BD Biosciences, San Jose, CA, USA), and the data were analyzed using the FlowJo program (De Novo Software, Glendale, CA, USA).

### 2.10. TUNEL Assay

Cells were plated (5 × 10^5^ cells/well) in 60 mm cell culture dishes and incubated for 18 h in a humidified incubator. After incubation, cells were transfected with control/IL13Rα2 siRNA or treated with DMSO (0.1%) control/the indicated treatment for 48 h. After transfection, the cells were fixed in 4% formaldehyde solution at 4 °C for 20 min. After fixation, the cells were permeabilized with 0.2% Triton X100 (Sigma). DNA strand breaks labeling was performed using a TUNEL assay kit (Promega, Madison, WI, USA). Nuclei were dyed with DAPI.

### 2.11. Annexin V Staining Analysis

Cells were plated (5 × 10^5^ cells/well) in 60 mm culture dishes and incubated for 18 h in a humidified incubator. After incubation, cells were transfected with control/IL13Rα2 siRNA or treated with DMSO (0.1%) control/the indicated treatment for 48 h. The cells were trypsinized and resuspended in annexin V-binding buffer. The percentage of apoptotic cells was evaluated by a FITC annexin V apoptosis detection kit I (BD Biosciences) with PI according to the manufacturer’s protocol. 1 × 10^4^ events were collected for each run. Cells were analyzed by a FACSCalibur (BD Biosciences), and FlowJo software (De Novo Software) was used to analyze the data.

### 2.12. Western Blotting Analysis

Cells were lysed in lysis buffer (RIPA buffer, Cell Signaling Technology, USA) containing protease and phosphatase inhibitors. Centrifugation (10,000× *g*, 4 °C, 10 min) was carried out, and protein lysates were separated on 10% NuPAGE pre-casting gels (Invitrogen) and transferred to nitrocellulose membranes (Bio-Rad Laboratories). The membranes were blocked with 3% defatted dry milk powder at room temperature for 1 h, and immunoblotting was performed with specific primary antibodies (overnight, 4 °C). Membranes were incubated with HRP-conjugated anti-mouse or anti-rabbit IgG in 3% defatted dry milk powder at room temperature for 1 h. Finally, the bands were detected using ECL solution (GE Healthcare, Chicago, IL, USA) and ChemiDoc system (Bio-Rad Laboratories).

### 2.13. Immunoprecipitation Analysis

Cells were lysed in lysis buffer (RIPA buffer, Cell Signaling Technology) containing protease and phosphatase inhibitors. Centrifugation (10,000× *g*, 4 °C, 10 min) was carried out, and protein lysates were separated. The protein lysates were incubated with a specific primary antibody by rotating at 4 °C overnight. After then, 20 μL of 50% protein A or G-agarose slurry (Santa Cruz) was added to the lysates and rotating for 2 h at 4 °C. Protein A or G-agaroses containing antigen–antibody complexes were collected and rinsed with PBS. Immunoprecipitants were analyzed by Western blotting.

### 2.14. JAK2 Kinase Inhibition Assay

Inhibitory activity of AZD1480 and telmisartan against JAK2 was evaluated by JAK2 kinase assay kit (BPS Bioscience, San Diego, CA, USA) and Glo-Max kinase assay kit (Promega). Briefly, according to the manufacturer’s instructions, recombinant JAK2 protein was incubated with the indicated concentration of AZD1480 or telmisartan, peptide substrate, and ATP for 30 min at 37 °C. After incubation, the reaction mixture was incubated with Glo-Max solution for 30 min at room temperature to stop the reaction. Then, the remaining ATP level in each reaction was measured by a microplate reader for luminescence (Bio-Rad Laboratories).

### 2.15. Statistical Analysis

The immunohistochemical staining score for IL13Rα2 in the RCC tissue sample was grouped into negative and positive cases with receiver operating characteristic curve analysis [22,23,24]. The cutoff point for IL13Rα2 immunostaining score to discriminate negative or positive cases was determined at the point that significantly estimates patients’ death from RCC. The cutoff point has the highest area under the curve in the receiver operating characteristic curve analysis. The survival analysis was conducted for cancer-specific survival (CSS) and relapse-free survival (RFS) through December 2013. The duration for CSS was calculated from the date of diagnosis to the date of the patient’s last contact or death. The event in CSS analysis was the death of patients from RCC. The death of patients from other causes or alive of patients finally contact was censored in CSS analysis. The duration for RFS was calculated from the date of diagnosis to the date of the last contact without relapse, the date of the first relapse, or patients’ death. The event in RFS analysis was a relapse of RCC or death of patients from RCC. Patients’ death from other causes or alive of patients finally contact without relapse were censored in RFS analysis. The survival analysis was performed with univariate and multivariate Cox proportional hazards regression analyses and Kaplan–Meier survival analysis using SPSS software (version 20.0, IBM, CA, USA). The association between clinicopathological factors was analyzed by Pearson’s chi-squared test using SPSS software, and all statistical tests were two-sided. The values of *P* lower than 0.05 were considered statistically significant.

## 3. Results

### 3.1. Immunohistochemical Expression of IL13Rα2 Is Associated with Poor Prognosis of RCC Patients

**Figure 1:**
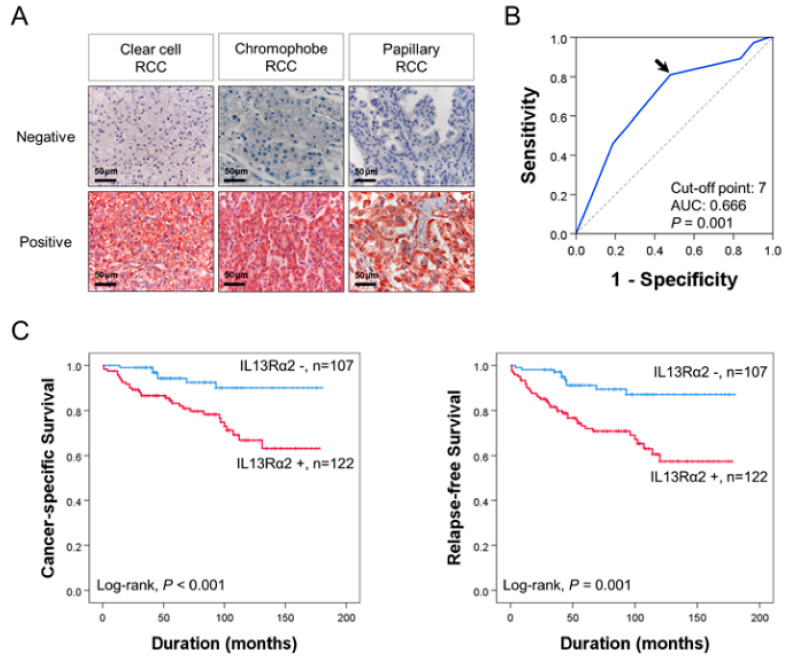
Immunohistochemical expression and survival analysis for the expression of IL13Rα2 in renal cell carcinomas. (**A**) Immunohistochemical expression of IL13Rα2 in clear cell renal cell carcinoma, chromophobe renal cell carcinoma, and papillary renal cell carcinoma tissue. Original magnification, ×400. (**B**) Receiver operator characteristic curve analysis to determine the cutoff point of IL13Rα2 immunostaining. The cutoff point is determined to predict cancer-specific survival of renal cell carcinoma patients. The cutoff point has the highest area under the curve (AUC). Arrow indicates a cutoff point for the IL13Rα2 immunostaining. (**C**) Kaplan–Meier survival analysis for cancer-specific survival and relapse-free survival according to the immunohistochemical positivity for IL13Rα2 in 229 cell renal cell carcinomas.

**Figure 2:**
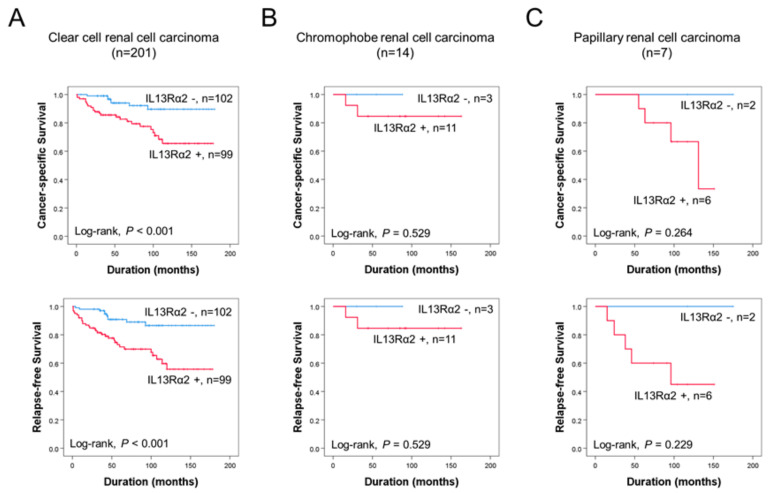
Kaplan–Meier survival analysis in histologic subtypes of renal cell carcinomas. Kaplan–Meier survival curves for cancer-specific survival (CSS) and relapse-free survival (RFS) according to the expression of IL13Rα2 in clear cell renal cell carcinoma (**A**), chromophobe renal cell carcinoma (**B**), and papillary renal cell carcinoma (**C**).

The immunohistochemical staining for IL13Rα2 was seen in tumor cells of all histologic subtypes of RCC (Figure 1A). The cut-off point for IL13Rα2 immunostaining was seven in receiver operating characteristic curve analysis (Figure 1B). The cases have immunohistochemical staining scores equal to, or greater than, seven were grouped as positive for IL13Rα2 staining. In this cut-off value, IL13Rα2-positivity was significantly associated with tumor size (*P* = 0.004), tumor stage (*P* = 0.002), histologic nuclear grade of tumor cells (*P* < 0.001), and histologic subtype of RCC (*P* = 0.005) in 229 cases of RCCs (Table 1). CCRCC is the major histologic subtype of RCC, and there were 201 cases of CCRCC in this study. Therefore, we also evaluated in CCRCC subgroup of RCCs. In CCRCC subgroup, IL13Rα2-positivity was significantly associated with tumor size (*P* = 0.005), tumor stage (*P* = 0.003), and histologic nuclear grade of tumor cells (*P* < 0.001) (Table 1). In 229 overall RCCs, the factors significantly associated with CCS or RFS in univariate analysis were age (CSS, *P* < 0.001; RFS, *P* = 0.005), tumor size (CSS, *P* < 0.001; RFS, *P* < 0.001), tumor stage (CSS, *P* < 0.001; RFS, *P* < 0.001), lymph node metastasis (CSS, *P* = 0.615; RFS, *P* < 0.001), histologic nuclear grade (CSS, overall *P* = 0.032; RFS, overall *P* = 0.008), tumor necrosis (CSS, *P* < 0.001; RFS, *P* = 0.004), and IL13Rα2-positivity (CSS, *P* = 0.002; RFS, *P* < 0.001) (Table 2). The IL13Rα2-positivity showed a 3.726-fold (95% confidence interval [95% CI]; 1.636–8.489, *P* = 0.002) greater risk of death and a 3.625-fold (95% CI; 1.806–7.278, *P* < 0.001) greater risk of relapse or death of RCC patients (Table 2). The Kaplan–Meier survival curves for CSS and RFS according to IL13Rα2-positivity in overall RCC are presented in Figure 1C. In 201 CCRCCs, the factors significantly associated with CCS or RFS in univariate analysis were age (CSS, *P* = 0.004; RFS, *P* = 0.012), tumor size (CSS, *P* < 0.001; RFS, *P* < 0.001), tumor stage (CSS, *P* < 0.001; RFS, *P* < 0.001), lymph node metastasis (CSS, *P* = 0.721; RFS, *P* = 0.011), histologic nuclear grade (CSS, overall *P* = 0.170; RFS, overall *P* = 0.028), tumor necrosis (CSS, *P* = 0.005; RFS, *P* = 0.063), and IL13Rα2-positivity (CSS, *P* = 0.003; RFS, *P* < 0.001) (Table 2). The IL13Rα2-positivity had a 3.591-fold (95% CI; 1.546–8.342, *P* = 0.003) greater risk of death from CCRCC and a 3.518-fold (95% CI; 1.724–7.181, *P* < 0.001) greater risk of relapse or death from CCRCC (Table 2). The Kaplan–Meier survival analysis also showed significant prognostic significance of IL13Rα2 expression for CSS and RFS in CCRCC subgroups (Figure 2A). However, in chromophobe RCC and papillary RCC, despite relatively shorter survival of IL13Rα2-positive subgroups compared with IL13Rα2-negative subgroups, there was no significant difference in survival of patients (Figure 2B,C). Multivariate analysis was performed with the factors significantly associated with CSS or RFS in univariate analysis. The factors included in multivariate analysis were age, tumor size, tumor stage, lymph node metastasis, histologic nuclear grade, tumor necrosis, and immunohistochemical expression of IL13Rα2. In 272 overall RCCs, age (CSS, *P* = 0.018), tumor stage (CSS, *P* = 0.005; RFS, *P* < 0.001), tumor necrosis (CSS, *P* = 0.005; RFS, *P* = 0.015), and IL13Rα2 expression (CSS, *P* = 0.025; RFS, *P* = 0.004) were significantly associated with CSS or RFS (Table 3). The IL13Rα2-positivity had a 2.627-fold (95% CI; 1.132–6.097) greater risk of death and a 2.801-fold (95% CI; 1.3795.688) greater risk of relapse or death of RCC patients (Table 3). In 201 CCRCCs, age (CSS, *P* = 0.042), tumor stage (CSS, *P* = 0.010; RFS, *P* < 0.001), tumor necrosis (CSS, *P* = 0.006; RFS, *P* = 0.054), and IL13Rα2 expression (CSS, *P* = 0.019; RFS, *P* = 0.005) were significantly associated with CSS or RFS (Table 3). The IL13Rα2-positivity showed a 2.792-fold (95% CI; 1.182–6.595) greater risk of death and a 2.838-fold (95% CI; 1.372–5.870, *P* < 0.001) greater risk of relapse or death of CCRCC patients (Table 3). Taken together, we investigated the clinicopathologically significant oncogenic role of IL13Rα2 in 229 RCC patients and the high expression of IL13Rα2 was significantly associated with cancer-specific survival and relapse-free survival in univariate and multivariate analysis.

### 3.2. Knockdown of IL13Rα2 Displays the AntiProliferative Activity in A498, ACHN, Caki1, and Caki2 Cells

**Figure 3:**
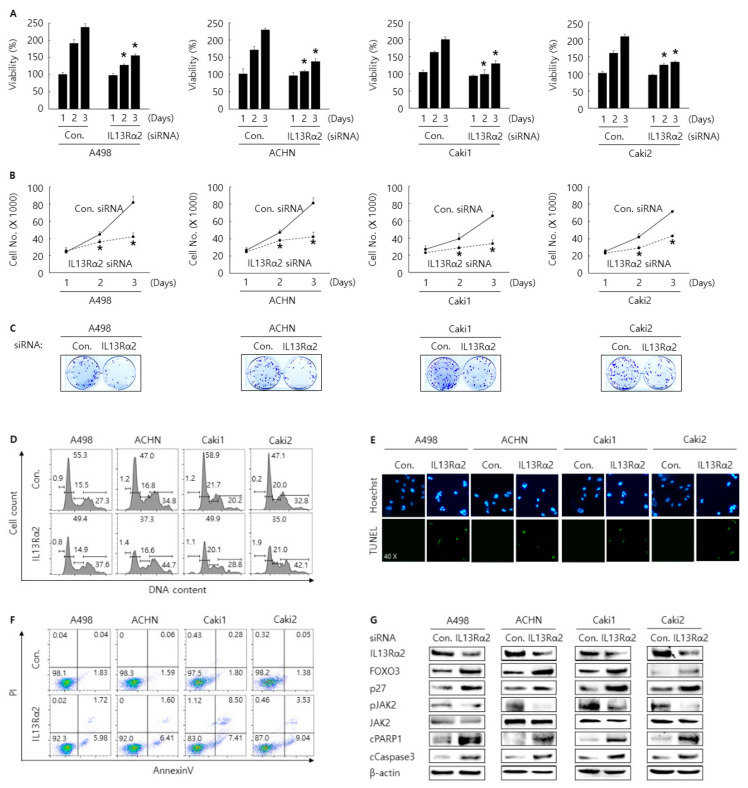
Antiproliferative effect by transfection of siRNA against IL13Rα2 in A498, ACHN, Caki1, and Caki2 cells. Cell viability and proliferation rate were determined by WST-1 (**A**), cell counting assay (**B**) for 24, 48, and 72 h, and Colony formation assay for 14 days (**C**). This result is representative data of at least three independent experiments, and the error bar indicates mean ± standard error (STE). * stands for the *P-*value < 0.05. Cell cycle arrest for 48 h after transfection was determined by cell cycle analysis (**D**). Apoptosis for 48 h after transfection was determined by Annexin V staining analysis (**E**) and Terminal deoxynucleotidyl transferase dUTP nick end labeling (TUNEL) assay (**F**). This result represents at least three independent experiments (**G**) Western blotting analysis of proteins related to cell cycle arrest and apoptosis for 48 h after transfection. β-actin was used for a gel-loading control.

In 229 cases of human RCC, a significant association between the expression IL13Rα2 and poor prognosis was observed by tissue microarray. Hence, as the next step, we tried to investigate the possible oncogenic role of IL13Rα2 by performing in vitro assays for cell proliferation, cell cycle arrest, and apoptosis in RCC cells after transfection of siRNA against IL13Rα2. WST-1 and cell counting assay were conducted to evaluate the antiproliferative activity of the knockdown of IL13Rα2. Cells were transfected with control or siRNA against IL13Rα2 and incubated for the indicated time. As shown in Figure 3A,B, compared to the control, cells transfected with siRNA against IL13Rα2 showed a decreased proliferation rate, which was confirmed by performing colony formation assay (Figure 3C). Cell cycle analysis showed that knockdown of IL13Rα2 with siRNA increased G2/M population in A498, ACHN, Caki1, and Caki2 cells compared to control siRNA (Figure 3D). TUNEL and annexin V staining assay results showed that knockdown of IL13Rα2 with siRNA increased the apoptosis in A498, ACHN Caki1, and Caki2 cells compared to control siRNA (Figure 3E,F). Western blotting analysis indicated that knockdown of IL13Rα2 with siRNA increased the expression of cleaved PARP1, cleaved caspase3, FOXO3, and p27 (Figure 3G). Overall, these results indicate that knockdown of IL13Rα2 with siRNA transfection could regulate proliferation, cell cycle arrest, and apoptosis in A498, ACHN, Cak1, and Caki2 RCC cells.

### 3.3. Knockdown of IL13Rα2 Attenuates the Protein Interaction Among IL13Rα2, pJAK2, and FOXO3 in A498, ACHN, Caki1, and Caki2 Cells

**Figure 4:**
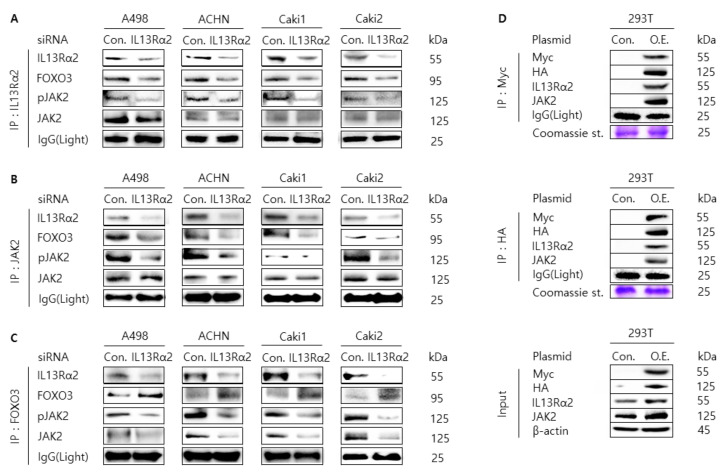
Protein interaction between IL13Rα2 and JAK2. Knock-down of IL4Rα2 in A498, ACHN, Caki1, and Caki2 cells reduced the interaction between IL13Rα2 and JAK2. Cells were transfected with siRNA against IL4Rα2 or control siRNA. Then cell lysates were immunoprecipitated with antibodies against IL4Rα2 (**A**), JAK2 (**B**), or FOXO3 (**C**). The immunoprecipitated proteins were immunoblotted by IL4Rα2, pJAK2, JAK2, and FOXO3 antibodies. Light chain of IgG was used for the loading control. (**D**) 293T cells were co-transfected with Myc-IL4Rα2 and HA-JAK2 (O.E.) or a control plasmid DNA (pCMV6-C-Myc-Flag and pCMV3-C-HA, Con.) as indicated. Then cell lysates were immunoprecipitated with antibodies against Myc or HA. The immunoprecipitated proteins were immunoblotted by Myc, HA, IL4Rα2, JAK2 antibodies. Light chain of IgG and Coomassie Blue staining of SDS–PAGE were used for the loading control.

In the previous report, we found that pJAK2 interacts with FOXO3, which was regulated by type II IL4R and IL13Rα1 heterodimeric receptor complex [21]. Since IL13Rα2 can accept IL13 as the same ligand with type II IL4R and IL13Rα1 complex, we examined whether the phosphorylation level of JAK2 was regulated by knockdown of siRNA against IL13Rα2 in RCC cells. When A498, ACHN, Caki1, Caki2, and 293T cell lysates were analyzed by Western blotting for IL13Rα2, pJAK2, JAK2, and FOXO3, there seemed the correlation pattern between IL13Rα2 and pJAK2 except for Caki2 cell lysates (Supplementary Figure S1A). In contrast, the expression of pJAK2 and FOXO3 was reversely correlated. In addition, as shown in Figure 3G, the expression of pJAK2 was significantly downregulated by transfection of IL13Rα2 with siRNA in A498, ACHN Caki1, and Caki2 cells compared to control siRNA. Then, to investigate the protein interaction among IL13Rα2, pJAK2, and FOXO3, we performed co-immunoprecipitation experiments with an antibody against IL13Rα2, JAK2, and FOXO3 followed by immunoblot analysis with an antibody against IL13Rα2, pJAK2, JAK2, and FOXO3 in A498, ACHN Caki1, and Caki2 cells transfected with siRNA against IL13Rα2. As shown in Figure 4A–C, the protein interaction among IL13Rα2, pJAK2 and FOXO3 was weakened in RCC cells transfected with siRNA against IL13Rα2 compared to the control siRNA. Furthermore, we could observe that the level of protein interaction between IL13Rα2 and JAK2 was increased in 293T cells co-transfected with overexpression plasmid DNA for IL13Rα2 or JAK2 (Figure 3D). Collectively, these results implicate that IL13Rα2 interacts with JAK2, which may regulate the protein expression level of FOXO3.

### 3.4. Telmisartan Suppresses Cell Proliferation and Induces Apoptosis and Cell Cycle Arrest in A498, ACHN, Caki1, and Caki2 Cells Via Inhibition of JAK2

**Figure 5:**
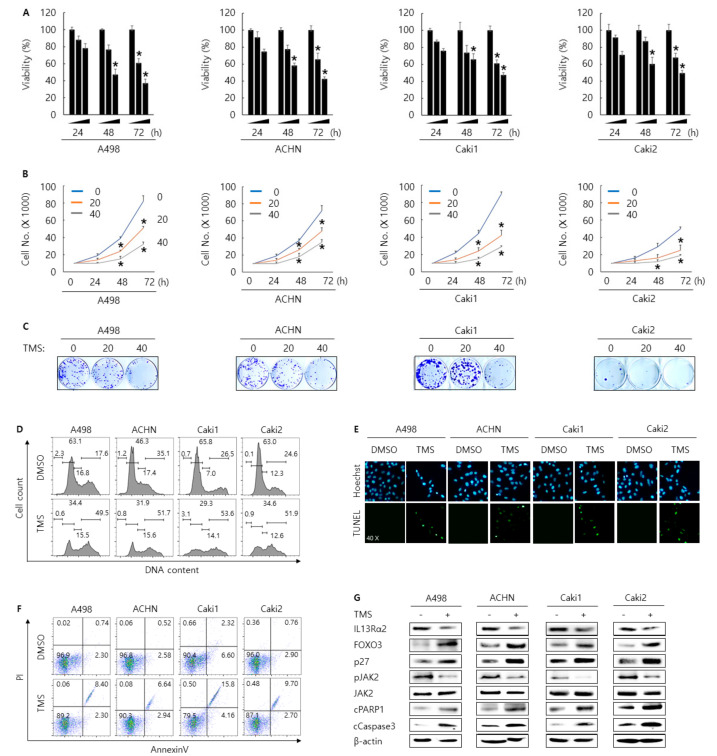
Antiproliferative effect by telmisartan treatment in A498, ACHN, Caki1, and Caki2 cells. Cell viability and proliferation rate were determined by WST-1 (**A**), cell counting assay (**B**) for 24, 48, and 72 h, and Colony formation assay for 14 days (**C**) after treatment of telmisartan (0, 20, and 40 μM). This result is representative data of at least three independent experiments, and the error bar indicates mean ± standard error (STE). * stands for the *P-*value < 0.05. Cell cycle arrest for 48 h after treatment of telmisartan (40 μM) was determined by cell cycle analysis (**D**). Apoptosis for 48 h after treatment of telmisartan (40 μM) was determined by Annexin V staining analysis (**E**) and Terminal deoxynucleotidyl transferase dUTP nick end labeling (TUNEL) assay (**F**). This result is representative data of at least three independent experiments. (**G**) Western blotting analysis of proteins related to cell cycle arrest and apoptosis for 48 h after treatment of telmisartan (40 μM). β-actin was used for a gel-loading control.

Previously, we reported that type II IL4Rα and IL13Rα1 complex are involved in RCC progress through regulation JAK2/FOXO3 pathway [21]. In addition, in this study, we showed that JAK2 was regulated by IL13Rα2. Thus, we thought that JAK2 was the common downstream-signaling kinase under the type II IL4Rα and IL13Rα1 complex and IL13Rα2. Hence, we tried to find the novel chemical inhibitor against JAK2 as the therapeutic way to treat RCC by screening an FDA-approved drug library (821 drugs) with a JAK2 kinase assay kit. After narrowing down the possible candidates, telmisartan, a clinically used medicine against hypertension, could be selected as one of the strongest JAK2 inhibitors from 821 drugs. As shown in Supplementary Figure S1B, telmisartan reduced ATP consumption in a dose-dependent manner in vitro. In fact, telmisartan treatment decreased the phosphorylation level of JAK2 in A498, ACHN, and 293T transfected with JAK2 overexpression plasmid DNA (Supplementary Figure S1C). To determine the anti-carcinogenic effect of telmisartan, we conducted in vitro assays for cell proliferation, cell cycle arrest, and apoptosis in RCC cells after telmisartan treatment. Cells were treated with 0, 20, and 40 µM of telmisartan and incubated for the indicated time. As shown in Figure 5A–C, telmisartan treatment decreased cell proliferation rate in a dose and time-dependent manner. We found that telmisartan treatment increased the G2/M population in A498, ACHN, Caki1, and Caki2 cells compared to DMSO control (Figure 5D). TUNEL and annexin V staining assay results showed that telmisartan treatment increased the apoptosis in A498, ACHN Caki1, and Caki2 cells compared to DMSO control (Figure 5E,F). Western blotting analysis indicated that telmisartan increased the expression of cleaved PARP1, cleaved caspase3, FOXO3, and p27, whereas decreased the expression of IL13Rα2 and pJAK2 (Figure 5G). Overall, these results indicate that telmisartan treatment could regulate proliferation, cell cycle arrest, and apoptosis in A498, ACHN, Cak1, and Caki2 RCC cells via inhibition of JAK2.

## 4. Discussion

It has been reported that IL13Rα2 was overexpressed in various cancers, such as glioblastoma, metastatic colorectal cancer, and ovarian cancer, which suggests that IL13Rα2 can play crucial roles in the development of various cancer types [25,26]. A recent study revealed that IL13Rα2 might be an important therapeutic target in a perineural invasion, the invasion of cancer to nerves [27]. In another study, IL13Rα2 was closely related to cancer cell migration, which indicated that IL13Rα2 might be a key factor in metastasis in cancers [28]. It was reported that IL13Rα2 was a functional receptor-mediating-signaling pathway in human pancreatic cancer cell lines [29]. They showed that IL13 induced the activation of transforming growth factor-β (TGFβ) through the AP-1 pathway, which can promote tumorigenesis caused by immunosuppression. In another study using the mouse model, it was demonstrated that two kinds of humanized scFv based chimeric antigen receptor (CAR) T cells targeting IL13Rα2 inhibited tumor growth in vitro and in vivo [30]. Sunitinib is an agent for treating metastatic or unresectable clear cell RCC, and IL13Rα2 can be a potential target to overcome sunitinib resistance [31]. However, the exact mechanism related to IL13Rα2 has not been investigated in RCC development. As shown in Figure 1 and Figure 2, immunohistochemical expression of IL13Rα2 was highly associated with cancer-specific survival and relapse-free survival by univariate and multivariate analysis in 229 RCC patients. In addition, the oncogenic role of IL13Rα2 was confirmed by the in vitro cell assay. Knock-down of IL13Rα2 showed the antiproliferative activity in A498, ACHN, Caki1, and Caki2 cells (Figure 3). As shown in Figure 3G, the expression of pJAK2 was significantly downregulated by transfection of IL13Rα2 with siRNA in RCC cells. Mechanistically, IL13Rα2 seemed to interact with JAK2 in RCC cells to activate the phosphorylation of JAK2, which may downregulate FOXO3, a representative tumor-suppressive transcriptional factor. To the best of our knowledge, this is the first research to demonstrate the IL13Rα2/JAK2/FOXO3-signaling pathway in cancer development.

Atopic dermatitis (AD) has been the most common type of chronic inflammatory skin disease [32]. JAK2 inhibitors have been identified as effective reagents for the treatment of atopic dermatitis [33]. A recent study showed that JTE-052, which is a novel JAK inhibitor suppressed skin inflammation and had therapeutic effects on chronic dermatitis in rodent models [34]. Interestingly, the recent clinical report has shown that cream containing ruxolitinib that is JAK1/JAK2 inhibitor alleviated AD symptoms and itch effectively in AD patients [35]. These studies suggested that JAK2 inhibitor could be a promising reagent for developing effective drugs for AD treatment. Furthermore, JAK2 inhibitor has been considered a promising therapeutic reagent for arthritis treatment [36]. A recent study has reported that ferulic acid showed anti-arthritic activity in rats induced arthritis through inhibition of the JAK/STAT pathway [37]. It was also reported that the Ershiwuwei Lvxue pill (ELP) that is Tibetan traditional medicine, reduced collagen-induced arthritis through JAK2/STAT3-signaling pathway inhibition [38]. These studies indicated that JAK2 inhibitor also could be considered an effective reagent for arthritis treatment.

IL-13 has been known as a crucial cytokine in chronic airway inflammation, and it plays an important role in AD pathogenesis [39,40]. Because IL-13 is a pivotal cytokine involved in allergic responses, it is important to find an effective way to alleviate immune responses by inhibiting IL-13 [41]. A recent study demonstrated that inhibition of IL-13 for AD is a new pathway, which suggested that IL-13 inhibitors could be an effective reagent for AD treatment [42]. It was reported that lebrikizumab is an IL-13 inhibitor that has the potential to treat moderate-to-severe AD with fewer side effects [43]. A clinical report showed that tralokinumab is the other IL-13 inhibitor that shows promising results of alleviating moderate-to-severe AD in adult patients. In short, these results supported that IL-13 inhibitor appears to have the potential to be a promising reagent for the development of new drugs for AD treatment.

Janus kinases, often referred as JAK, have been known as cytoplasmic tyrosine kinase combined with intracellular domains of various cytokine receptors [44]. JAK family member is divided into JAK1, JAK2, JAK3, and TYK2 [45]. According to recent studies, JAK2/STAT3 signaling pathway played critical roles in metastasis and progression of cancers, which implied that JAK2 might be a crucial therapeutic target for treatment of cancer [46,47,48,49]. Recent study showed that salidroside had anti-cancer effects and suppressed RCC proliferation through inhibition of JAK2/STAT3 signaling pathway [50]. The data presented in this study indicated that salidroside decreased the levels of phosphorylated STAT3 and JAK2 in A498 and 786-0 RCC cells. It was also reported that thymoquinone, a natural compound extracted from black seed oil, possessed anti-cancer effects in RCC cells [51]. According to them, inhibition of JAK2/STAT3 signaling pathway was observed after treatment of thymoquinone in Caki2 cells. Furthermore, recent studies have reported that the synthetic JAK2 inhibitor was considered as the therapeutic agent for other cancer types [52,53,54]. It was reported that treatment of JAK inhibitors CEP-33779 and NVP-BSK805 helped vincristine work effectively by sensitizing drug-resistant KBV20C oral cancer cells [55]. AG490, JAK2 inhibitor, also inhibited the proliferation and invasion of gallbladder cancer cells through inhibition of JAK2/STAT3 signaling pathway [54]. Thus, our current study supported that JAK2 has a potential to be an important target for various cancer treatment.

Telmisartan is angiotensin II receptor blocker and selectively inhibits the binding of angiotensin II into AT1 receptor [56]. Telmisartan was approved by FDA in 1998 and it has been used to treat high blood pressure and heart failure [57,58,59]. It also has been reported that telmisartan has anti-cancer effect against several cancer cell lines [60,61,62]. Recent study showed that telmisartan has cytotoxic effect through generation of reactive oxygen species (ROS) and upregulation of death receptor 5 (DR5) in human lung cancer A549 cells [63]. It was reported that telmisartan inhibited cancer cell growth and induced DNA damage in HHUA human endometrial cancer cells [64]. In another study, telmisartan downregulated Bcl-2 and induced apoptosis in 786-0 RCC cells [65]. Also, recent study has shown that telmisartan exhibited anti-cancer effect in MKN74 gastric cancer cells in vitro and in vivo [66]. Interestingly, this study showed that telmisartan inhibited tumor growth through cell cycle arrest in a mouse xenograft model of gastric cancer. Furthermore, growth inhibitory effect of telmisartan was observed in esophageal squamous cell carcinoma xenograft mouse model [67]. Similar with the previous studies, we observed that telmisartan treatment suppressed cell proliferation and induced cell cycle arrest and apoptosis via inhibition of JAK2 in human RCC cells. However, we still need to perform in vivo experiments using RCC mouse model to prove the anti-cancer activity of telmisartan. We selected telmisartan as one of the strongest JAK2 inhibitors from 821 FDA approved drugs. Since we adopted the screening way based on the assay to measure ATP consumption by JAK2, we thought that telmisartan might compete with ATP to bind the ATP binding site in JAK2. We are planning to conduct the competitive enzyme assay and simulate in silico docking model to prove this hypothesis. As shown in Figure 5G, interestingly, telmisartan treatment caused the downregulation of IL13Rα2. It seems that inhibition of JAK2 by telmisartan might induce the transcriptional downregulation of IL13Rα2 through inhibition of the phosphorylation of STAT3 transcriptional factor. So, we plan to perform other experiments demonstrating that STAT3 bind to the promoter region of IL13Rα2 and whether the binding affinity of STAT3 on the promoter region was weakened by JAK2 inhibition or not.

Since telmisartan has been used to treat heart disease for 22 years, there are lots of previous reports for researcher to examine the possible working mechanism of telmisartan in terms of anti-cancer activity. The relationship between JAK2 and angiotensin II signaling pathway has been investigated in various studies [68,69,70,71]. It has been reported that angiotensin II activates STAT3 through the IL6/gp130/JAK2 signaling pathway in cardiomyocytes [72]. AG490, well-known JAK2 inhibitor, inhibited angiotensin II-induced differentiation of bone marrow-derived mesenchymal stem cells (BM-MSCs) into keratinocytes, which suggested that JAK2 is associated with angiotensin II signaling pathway [73]. Recent study has shown that angiotensin II upregulated nitroxidative stress via JAK2/STAT3 signaling pathway leading to the hyperproliferation of vascular smooth muscle cells (VSMCs) [74]. In another study, it is demonstrated that inhibition of angiotensin II through JAK2/STAT3 signaling pathway suppressed tubular epithelial myofibroblast trans-differentiation mediated by hepatocyte growth factor (HGF) [75]. Thus, we thought that blocking of angiotensin II binding into AT1 receptor by telmisartan might cause the inhibition of JAK2 through direct or indirect signaling pathway in RCC cells. We might need to investigate the change of the phosphorylation status of JAK2 under knock-down of AT1 receptor in RCC cells. Peroxisome proliferator-activated receptor γ (PPARγ) is also well-known agonistic target of telmisartan. PPARγ is a member of nuclear receptor family and it plays an important role in regulating lipid metabolism [76]. According to a previous research, activation of JAK2/STAT3 signaling pathway was associated with downregulation of PPARγ, which promoted fibrosis in rats [77]. Furthermore, PPARγ decreased the protein expression of suppressor of cytokine signaling 3 (SOCS3) through inhibition of JAK2/STAT3 signaling pathway leading to alleviation of hepatocyte steatosis [78]. Additionally, it was reported that pioglitazone, one of PPARγ agonists, inhibited breast cancer growth by regulating JAK2/STAT signaling pathway in vitro and in vivo [79]. For the further study, we are trying to examine that rosiglitazone, FDA approved hypoglycemic agent as PPARγ agonists, has anti-cancer activity against RCC through inhibition of JAK2 phosphorylation.

**Figure 6:**
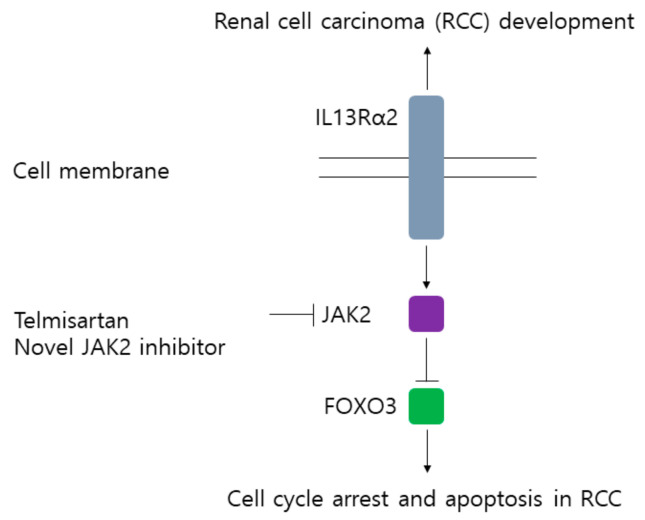
A diagram for the possible oncogenic role of IL13Rα2 in renal cell carcinoma (RCC) by activation of JAK2 and inhibition of FOXO3.

In this study, we demonstrated the clinicopathologically significance of IL13Rα2, a kind of the independent receptor for IL13, in RCC progression. Mechanistically, downregulation of IL13Rα2 in RCC cells seemed to decrease the phosphorylation of JAK2 and increase expression of FOXO3, suggesting that IL13Rα2 probably is involved in the progression of RCC through JAK2/FOXO3 pathway (Figure 6). In addition, we screened an FDA approved drug library to identify the novel candidates inhibiting JAK2 in RCC cells and selected telmisartan as the one of strongest JAK2 inhibitors. Telmisartan displayed the anti-proliferative activity in RCC cells, which could be one of the therapeutic options for RCC patients.

## Figures and Tables

**Table 1:** Clinicopathologic variables and the expression status of IL13Rα2 in renal cell carcinomas.

Characteristics		Overall Renal Cell Carcinoma (*n* = 229)			Clear Cell Renal Cell Carcinoma (*n* = 201)		
		No.	IL13Rα2 Positive	*P*	No.	IL13Rα2 Positive	*P*
Sex	Male	156	86 (55%)	0.411	140	71 (51%)	0.530
	Female	73	36 (49%)		61	28 (46%)	
Age, y	≤55	95	46 (48%)	0.215	82	35 (43%)	0.122
	>55	134	76 (57%)		119	64 (54%)	
Tumor size, cm	≤7	193	95 (49%)	0.004	169	76 (45%)	0.005
	>7	36	27 (75%)		32	23 (72%)	
TNM stage	I	183	88 (48%)	0.002	163	72 (44%)	0.003
	II-IV	46	34 (74%)		38	27 (71%)	
LN metastasis	Absence	226	119 (53%)	0.103	199	97 (49%)	0.149
	Presence	3	3 (100%)		2	2 (100%)	
Nuclear grade	1	45	17 (38%)	<0.001	36	10 (28%)	<0.001
	2	134	67 (50%)		123	59 (48%)	
	3 and 4	50	38 (76%)		42	30 (71%)	
Necrosis	Absence	196	102 (52%)	0.362	174	85 (49%)	0.772
	Presence	33	20 (61%)		27	14 (52%)	
Histologic type	Clear cell	201	99 (49%)	0.005			
	Chromophobe	16	13 (81%)				
	Papillary	12	10 (83%)				

**Table 2:** Univariate Cox regression analysis of cancer-specific survival and relapse-free survival in renal cell carcinoma patients.

Characteristics.	No.	CSS		RFS	
		HR (95% CI)	*P*	HR (95% CI)	*P*
Overall RCC (*n* = 229)					
Sex, male (vs. female)	156/229	0.564 (0.258–1.234)	0.152	0.513 (0.255–1.030)	0.060
Age, y, >55 (vs. ≤55)	134/229	4.386 (1.828–10.524)	<0.001	2.537 (1.319–4.880)	0.005
Tumor size, >7 cm (vs. ≤7 cm)	36/229	3.415 (1.736–6.715)	<0.001	3.984 (2.218–7.155)	<0.001
TNM stage, I (vs. II-IV)	46/229	4.231 (2.219–8.068)	<0.001	5.166 (2.930–9.018)	<0.001
LN metastasis, presence (vs. absence)	3/229	1.670 (0.226–12.308)	0.615	17.410 (3.874–78.249)	<0.001
Nuclear grade, 1	45/229	1	0.032	1	0.008
2	134/229	0.943 (0.347–2.564)	0.909	1.172 (0.476–2.883)	0.730
3 and 4	50/229	2.327 (0.836–6.476)	0.106	2.846 (1.128–7.179)	0.027
Necrosis, presence (vs. absence)	33/229	3.620 (1.842–7.114)	<0.001	2.542 (1.345–4.807)	0.004
Histologic type, clear cell	201/229	1	0.654	1	0.328
chromophobe	16/229	0.808 (0.193–3.382)	0.771	0.585 (0.141–2.421)	0.460
papillary	12/229	1.570 (0.553–4.462)	0.397	1.802 (0.711–4.565)	0.214
IL13Rα2, positive (vs. negative)	122/229	3.726 (1.636–8.489)	0.002	3.625 (1.806–7.278)	<0.001
Clear cell RCC (*n* = 201)					
Sex, male (vs. female)	140/201	0.541 (0.222–1.319)	0.177	0.523 (0.241–1.132)	0.100
Age, y, >55 (vs. ≤55)	119/201	4.152 (1.593–10.822)	0.004	2.491 (1.220–5.084)	0.012
Tumor size, >7 cm (vs. ≤7 cm)	32/201	3.977 (1.928–8.204)	<0.001	4.773 (2.560–8.900)	<0.001
TNM stage, I (vs. II-IV)	38/201	3.964 (1.953–8.049)	<0.001	5.199 (2.814–9.604)	<0.001
LN metastasis, presence (vs. absence)	2/201	0.049 (0.000–7.516 × 10^5^)	0.721	14.681 (1.841–117.039)	0.011
Nuclear grade, 1	36/201	1	0.170	1	0.028
2	123/201	1.028 (0.344–3.075)	0.961	1.122 (0.423–2.978)	0.817
3 and 4	42/201	2.111 (0.661–6.739)	0.207	2.655 (0.955–7.380)	0.061
Necrosis, presence (vs. absence)	27/201	3.044 (1.401–6.617)	0.005	2.016 (0.962–4.225)	0.063
IL13Rα2, positive (vs. negative)	99/201	3.591 (1.546–8.342)	0.003	3.518 (1.724–7.181)	<0.001

Abbreviations: CSS, cancer-specific survival; RFS, relapse-free survival; HR, hazard ratio; 95% CI, 95% confidence interval; RCC, renal cell carcinoma; LN, lymph node.

**Table 3:** Multivariate Cox regression analysis of cancer-specific survival and relapse-free survival in renal cell carcinoma patients.

Characteristics	CSS		RFS	
	HR (95% CI)	*P*	HR (95% CI)	*P*
Overall RCC (*n* = 229) *				
Age, y, >55 (vs. ≤55)	2.941 (1.200–7.209)	0.018		
TNM stage, I (vs. II-IV)	2.600 (1.331–5.077)	0.005	4.036 (2.260–7.209)	<0.001
Necrosis, presence (vs. absence)	2.686 (1.350–5.345)	0.005	2.240 (1.172–4.278)	0.015
IL13Rα2, positive (vs. negative)	2.627 (1.132–6.097)	0.025	2.801 (1.379–5.688)	0.004
Clear cell RCC (*n* = 201) **				
Age, y, >55 (vs. ≤55)	2.779 (1.036–7.453)	0.042		
TNM stage, I (vs. II-IV)	2.616 (1.255–5.451)	0.010	4.214 (2.257–7.867)	<0.001
Necrosis, presence (vs. absence)	3.002 (1.361–6.618)	0.006	2.088 (0.988–4.414)	0.054
IL13Rα2, positive (vs. negative)	2.792 (1.182–6.595)	0.019	2.838 (1.372–5.870)	0.005

Abbreviations: CSS, cancer-specific survival; RFS, relapse-free survival; HR, hazard ratio; 95% CI, 95% confidence interval; RCC, renal cell carcinoma. * The variables included in the multivariate analysis were age, tumor size, tumor stage, histologic nuclear grade, tumor necrosis, and the expression of IL13Rα2. ** The variables included in the multivariate analysis were age, tumor size, tumor stage, histologic nuclear grade, tumor necrosis, and the expression of IL13Rα2.

## Data Availability

The datasets used in the current study are available from the corresponding author upon reasonable request.

## Supplementary Materials

The following are available online at https://www.mdpi.com/article/10.3390/jpm11040284/s1, Figure S1: (A). The correlation pattern between expression of IL13Rα2, pJAK2, JAK2, and FOXO3 in A498, ACHN, Caki1, and Caki2 cells. Western blotting analysis of IL13Rα2, pJAK2, JAK2, and FOXO3 in each cell line. β-actin was used for a gel-loading control; (B) Reduction of ATP consumption by telmisartan in a dose-dependent manner in vitro. JAK2 protein was incubated with the indicated concentration of AZD1480 or telmisartan, peptide substrate, and ATP for 30 min at 37 °C. After incubation, the reaction mixture was incubated with Glo-Max solution for 30 min at room temperature to stop the reaction. Then, the remaining ATP level in each reaction was measured by a microplate reader for luminescence; (C). Reduction of phosphorylation of JAK2 by telmisartan. Western blotting analysis of pJAK2 and JAK2 after telmisartan treatment (0, 10, 20, and 40 μM). β-actin was used for a gel-loading control.
