# Supplementary material for: IL13Rα2 Is Involved in the Progress of Renal Cell Carcinoma through the JAK2/FOXO3 Pathway
Source: J Pers Med. 2021 Apr 8;11(4):284. doi: 10.3390/jpm11040284 (PMC8068290; doi:10.3390/jpm11040284)
Supplement: Supplementary file 1 [file jpm-11-00284-s001.pdf]

IL13Rα2 Is Involved in the Progress of Renal Cell Carcinoma through JAK2/FOXO3 pathway

Mi-Ae Kang[1] †, Jongsung Lee[2] †, Chang Min Lee[3]†, Ho Sung Park[4, 5, 6], Kyu Yun Jang[4, 5, 6] *, and See-Hyoung Park[3] *

1. Department of Biological Science, Gachon University, Seongnam 13120, Republic of Korea.

2. Department of Integrative Biotechnology, Sungkyunkwan University, Suwon 16419, Republic of Korea.

3. Department of Bio and Chemical Engineering, Hongik University, Sejong 30016, Republic of Korea.

4. Department of Pathology, Jeonbuk National University Medical School, Jeonju, Republic of Korea.

5. Research Institute of Clinical Medicine of Jeonbuk National University, Jeonju, Republic of Korea.

6. Biomedical Research Institute of Jeonbuk National University Hospital, Jeonju, Republic of Korea.

† These authors contributed equally to this work.

* Corresponding authors

Kyu Yun Jang, Professor

Department of Pathology, Jeonbuk National University Medical School

Jeonju 54896, Republic of Korea

Tel: +82-63-270-3136

E-mail: kyjang@jbnu.ac.kr

See-Hyoung Park, Associate Professor

Department of Bio and Chemical Engineering, Hongik University

Sejong 30016, Republic of Korea

Tel: +82-44-860-2126

E-mail: shpark74@hongik.ac.kr

Supplementary materials

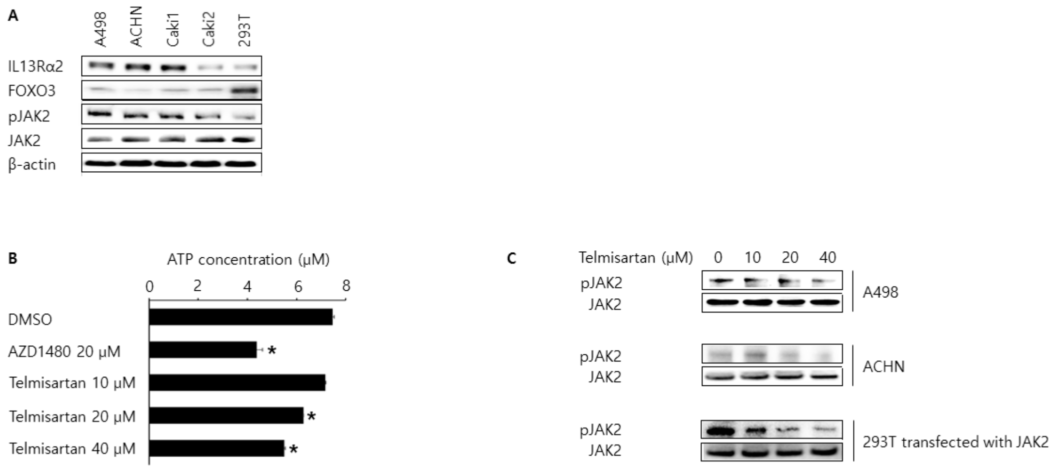

Supplementary Figure 1A. The correlation pattern between expression of IL13Rα2, pJAK2, JAK2, and FOXO3 in A498, ACHN, Caki1, and Caki2 cells. Western blotting analysis of IL13Rα2, pJAK2, JAK2, and FOXO3 in each cell lines. β-actin was used for a gel-loading control.

Supplementary Figure 1B. Reduction of ATP consumption by telmisartan with dose dependent manner in vitro. JAK2 protein was incubated with the indicated concentration of AZD1480 or telmisartan, peptide substrate, and ATP for 30 min at 37°C. After incubation, the reaction mixture was incubated with Glo-Max solution for 30 min at room temperature to stop the reaction. Then, the remaining ATP

level in each reaction was measured by a microplate reader for luminescence.

Supplementary Figure 1C. Reduction of phosphorylation of JAK2 by telmisartan. Western blotting analysis of pJAK2 and JAK2 after treatment of telmisartan (0, 10, 20, and 40 μM). β-actin was used for a gel-loading control.

Densitometry analysis for Figure 3G

	A498		ACHN		Caki1		Caki2	
	con	IL13Ra2	con	IL13Ra2	con	IL13Ra2	con	IL13Ra2
IL13Ra2	27849	15092	25516	10798	13722	7377	17877	6534
FOXO3	4929	11114	11304	25186	8067	15448	2028	7279
p27	11141	25411	18380	23165	6934	19233	15556	27775
pJAK2	4864	3734	24910	2127	23323	10691	12619	2367
JAK2	8706	5499	17097	14978	6978	4938	8963	6967
cPARP1	9621	28482	5429	20002	2034	9928	3001	17837
cCaspase3	5430	26097	2143	8214	2909	8244	11158	23602

Densitometry analysis for Figure 4A

	A498		ACHN		Caki1		Caki2	
	con	IL13Ra2	con	IL13Ra2	con	IL13Ra2	con	IL13Ra2
IL13Ra2	4148	2518	4738	1181	6606	2196	6230	1646
FOXO3	9065	5620	8287	3873	6831	3004	6271	2764
pJAK2	9375	4006	6781	3150	15579	3425	4599	2849
JAK2	15312	12676	8063	6139	9828	8376	9968	9757

Densitometry analysis for Figure 4B

	A498		ACHN		Caki1		Caki2	
	con	IL13Ra2	con	IL13Ra2	con	IL13Ra2	con	IL13Ra2
IL13Ra2	4363	1439	8691	3021	10279	4229	4092	1626
FOXO3	13010	8814	11157	3503	14281	3808	2734	1464
pJAK2	19243	5810	12932	6128	2470	866	22174	5842
JAK2	13820	11117	7381	6634	6011	5329	11098	9426

Densitometry analysis for Figure 4C

	A498		ACHN		Caki1		Caki2	
	con	IL13Ra2	con	IL13Ra2	con	IL13Ra2	con	IL13Ra2
IL13Ra2	8794	3948	9916	3443	14484	3772	7321	61
FOXO3	4271	8499	15489	24465	11932	17789	3983	12706
pJAK2	5878	1902	13775	6281	8573	4296	10475	1301
JAK2	9387	5392	5931	1449	5444	1518	10833	2379

Densitometry analysis for Figure 4D

	293T	
	Con.	O.E.
Myc	0	7113
HA	0	12712
IL13Ra2	0	7670
JAK2	0	12750
Myc	0	8036
HA	0	12005
IL13Ra2	0	5613
JAK2	0	7904
Myc	0	10466
HA	1036	21465
IL13Ra2	12170	21498
JAK2	16098	26307

Densitometry analysis for Figure 5G

	A498		ACHN		Caki1		Caki2	
TMS	-	+	-	+	-	+	-	+
IL13Ra2	24838	10880	26469	11222	7930	5788	5212	3027
FOXO3	5535	28413	13233	26459	4449	8373	1444	6318
p27	11429	23402	7119	17102	6283	11229	6272	11687
pJAK2	14754	2616	24944	10873	2991	610	8631	2775
JAK2	23313	25250	28190	21483	9734	10424	9387	7084
cPARP1	6932	20090	4413	7013	3382	7990	4982	12933
cCaspase3	2327	25089	2546	6647	687	4503	3815	10807

Supplementary table. Densitometry analysis for Figure 3G, 4A, 4B, 4C, 4D, and 5G. The relative density compared to the actin or IgG band was calculated by Image J program.
